# Paediatric eye care team: a comprehensive approach

**Published:** 2018-07-31

**Authors:** Rahul Ali

**Affiliations:** 1 Country Director: Orbis International, New Delhi, India.

**When India's capacity for paediatric eye care presented itself as a mammoth challenge, India Childhood Blindness Initiative (ICBI) was launched by Orbis to help ensure that India's children have access to quality eye care. These efforts have not only contributed towards building the capacity of various cadres of eye health professionals but have systematically created a milieu where paediatric ophthalmology could develop and flourish.**

**Figure:**
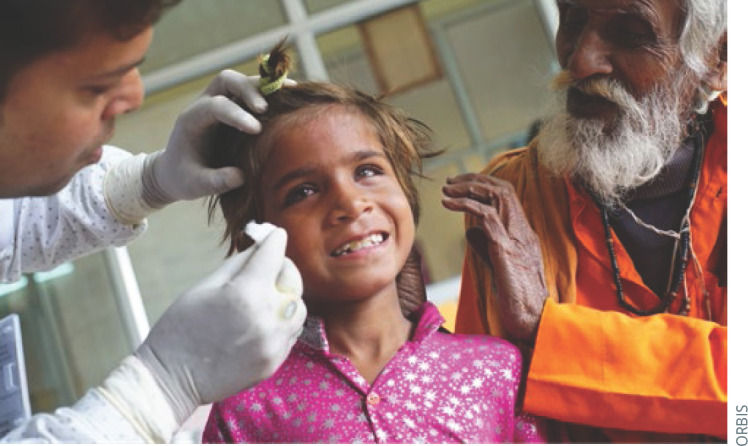
Examining children needs special skills and training. INDIA

One of the most critical deficits in global eye health is the lack of an adequately trained workforce. This is the very reason Orbis was founded: to provide ongoing training and support to eye care teams around the world.

India is home to more than 20 percent of the world's blind population and the largest number of blind children in any country. However, since children constitute only 3% of the world's blind population, childhood blindness has not been given its due importance as compared to other causes of blindness and visual impairment. In 2000, there were only four comprehensive tertiary paediatric eye care centers in India. At that time, with a population of 1 billion, India needed 100 Children's Eye Centers (CEC) as per the WHO guidelines of one center per 10 million population.

**Figure:**
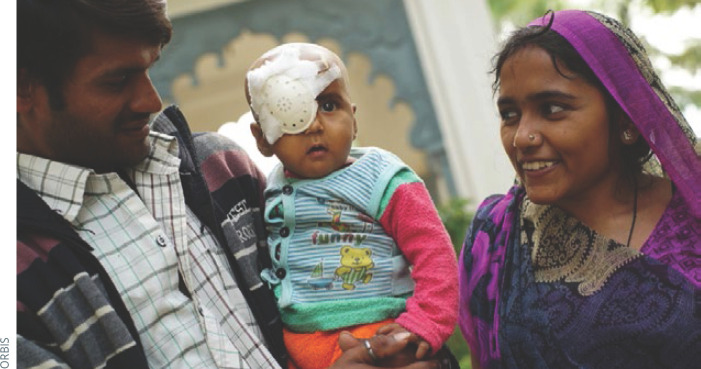
A paediatric ophthalmologist keeps in mind the nuances of a child's eye. INDIA

Building India's capacity for paediatric eye care presented itself as a mammoth challenge. Examining children needs special skills and their treatment protocols require specific training, knowledge and equipment. This meant we had to build the infrastructure for service delivery including equipping the facilities and supporting community work, along with development of all cadres of human resources required. Having the right people in the right place is the cornerstone of any successful public health programme. Keeping all of this in mind, in 2002, the India Childhood Blindness Initiative (ICBI) was launched by Orbis to help ensure that India's children have access to quality eye care for generations to come.

## Comprehensive capacity building

The programme began by identifying tertiary level eye hospitals where CECs could be established. Further, a country-wide survey was undertaken to generate evidence for human resource and infrastructure requirements for elimination of avoidable childhood blindness. This was the first time that such an extensive survey was undertaken in India.

The easier part was the development of infrastructure and systems. The challenging aspect was identifying staff and creating the paediatric ophthalmology teams at a time when paediatric ophthalmology was not recognised as a distinct subspecialty in India. This resulted in limited career options and therefore initially not many individuals were willing to undergo training.

## Paediatric ophthalmology team

It is imperative to take a team approach to paediatric ophthalmology to ensure comprehensive care. A paediatric eye care team typically comprises of at least six people: an ophthalmologist, anaesthesiologist, optometrist, nurse, counsellor and outreach coordinator who have undergone specific training in the management of eye diseases in children. Furthermore, other clinical, non-clinical and support staff are trained or oriented to complement the core team.

### Paediatric ophthalmologist

The ophthalmologist is trained to conduct a comprehensive ophthalmic evaluation of children keeping in mind the nuances of a child's eye. They need to be able to identify paediatric eye conditions and manage them appropriately to achieve the best possible outcome. The paediatric ophthalmologist will need to work closely with his/her colleagues in other departments - cornea, glaucoma, retina etc. -- and be aware of community paediatric eye care, vision screening and awareness initiatives.

### Anaesthesiologist

Unlike adults, children undergoing eye surgery will nearly always require general anaesthesia. A paediatric-trained anaesthesiologist makes it possible for children to safely undergo sight restoration surgeries, even a few days after birth.

### Optometrist

Optometrists are trained in the diagnosis and management of routine and complex eye conditions including refractive error, amblyopia (lazy eye), strabismus and more. They also travel outside the CEC to deliver services to children in the community.

### Paediatric nurse

Management of drugs, drawing blood, counselling patients and families, supervising sterilisation, managing the operating room, and assisting surgery are some of their responsibilities. Very often they become the child's best friend during the child's time at the hospital before, during and after a surgery.

### Counsellor

Patient education and counselling are an integral part of both medical and surgical management of a disease. In children, the eye is still developing therefore information on any intervention, especially surgery and care required before/after surgery are quite different as compared to adults. The counsellor assists parents in decision-making by giving detailed information about the management plan. They alleviate anxiety among parents by providing a detailed description of pre-operative evaluation including pre-anaesthesia check, post-operative care, discharge, and the necessity of long term follow-up. Their training includes basic anatomy and physiology of the eye, common eye diseases and their management, basics of surgical procedures and counselling skills including interpersonal communication.

### Community eye care/outreach coordinator

Planning, organising and reviewing outreach activities such as screening camps, community-based rehabilitation, school eye health programmes, etc. are taught. Networking with local government and building strategic relationships within the community are key to their role.

We strongly recommend organisations to have a comprehensive child protection policy to provide clear guidelines for them and their staff to ensure a safe environment for children.

For the team to work more effectively, we recommend:

- **Timing of training**

Considering varying durations of training programmes, it is important to plan the training such that all members complete their training and return to the new CEC around the same time to begin work as one team.

- **Training center**

All members should undergo training at the same institution to ensure ideological alignment and familiarity with the same systems and processes across the team.

## Developing the paediatric ophthalmology team

Once people were identified for training, ‘where’ and ‘how’ they would be trained continued to remain a challenge. To address this, three of the existing tertiary level paediatric facilities in the country were developed as paediatric ophthalmology learning and training centers (POLTCs) by providing infrastructure as well as technical support. This included standardisation of the curricula for different cadres of eye health professionals for the CECs and community work.

POLTCs offer fellowships in paediatric ophthalmology, short/long-term training programmes and periodically conducted workshops/refresher training as well as continuing medical education (CME). Conducting impactful research on child eye health is an integral part of a POLTC.

To aid continuing education and support, Orbis creates customised hands-on opportunities through the Flying Eye Hospital and hospital-based trainings (HBTs) to increase clinical and surgical abilities of eye care providers. These trainings are tailored to address the requirements of the trainee as well as the community they will be serving. HBTs are especially well-received as they provide an opportunity for the entire clinical staff to get trained and gain experience in their own setting.

**Figure:**
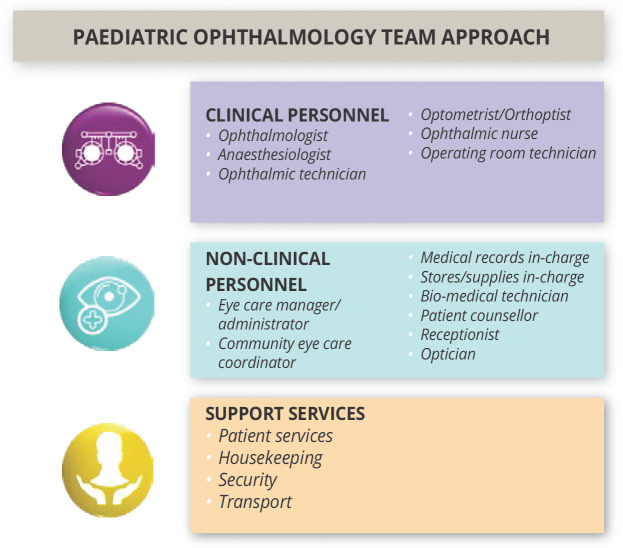


In addition, Cybersight, equal parts library, school and remote-medicine service, is open to all eye health professionals around the world for training, consultation and research. Also, it keeps professionals who have undergone training connected with their mentors.

These efforts have not only contributed towards building the capacity of various cadres of eye health professionals and their affiliated institutions to provide care and support to children in need but has systematically created a milieu where paediatric ophthalmology could develop and flourish as a distinct subspecialty within the Indian ophthalmology landscape.

Today there are 33 CECs that have been developed with Orbis support across 17 states in India, and the good work is continuing at these child-friendly facilities. POLTCs continue to provide training and support to the eye care system in India and many neighbouring countries. Further, this model has been successfully replicated in Nepal and Bangladesh.

